# Preparation of a Novel Nanofiltration Membrane and Study of Its Process for Removing Divalent Ions from Xinjiang Oilfield Wastewater

**DOI:** 10.3390/membranes16040151

**Published:** 2026-04-17

**Authors:** Zongneng Zheng, Di Liu, Jiahang Wan, Jianping Li, Kun Zhang, Yanxin Li, Haiyi Yang, Junwei Hou

**Affiliations:** 1 School of Petroleum Engineering, Karamay Vocational & Technical College, Karamay 834000, China; 2 State Key Laboratory of Heavy Oil Processing, China University of Petroleum (Beijing) at Karamay, Karamay 834000, China; 3 Karamay Xinkeao Petroleum Technological Co., Ltd., Karamay 834000, China

**Keywords:** hardness removal, oilfield wastewater, nanofiltration membrane, retention performance, membrane flux

## Abstract

The produced water from the No. 1 Oil Production Plant of Xinjiang Oilfield is rich in divalent ions, including Ca^2+^, Mg^2+^, and SO_4_^2−^, leading to extremely high scaling tendency that fails to meet the reinjection standard. Therefore, highly efficient water softening technology is urgently required for such wastewater treatment. In this study, a novel negatively charged nanofiltration (NF) membrane was fabricated via interfacial polymerization using 2-carboxypiperazine and trimesoyl chloride as monomers. The membrane was systematically characterized by scanning electron microscopy (SEM), X-ray photoelectron spectroscopy (XPS), and Fourier-transform infrared spectroscopy (FTIR), and its rejection performance was investigated under various conditions. Results show that the maximum rejection rates of the NF membrane reached 99% for SO_4_^2−^, 81% for Ca^2+^, and 94% for Mg^2+^, respectively. With increasing ion concentration, the removal efficiencies of Ca^2+^ and Mg^2+^ decreased, while that of SO_4_^2−^ increased slightly. Higher operating pressure significantly enhanced both ion removal and membrane flux, which was mainly attributed to the synergistic effects of Donnan electrostatic exclusion, membrane surface adsorption, and mass transfer resistance. When applied to treat real produced water from the No. 1 Oil Production Plant, the membrane achieved 100% removal of SO_4_^2−^, and 91% and 95% removal of Ca^2+^ and Mg^2+^, respectively. The scaling tendency of the treated effluent was completely eliminated. This work provides theoretical and technical support for the engineering application of nanofiltration technology in oilfield wastewater treatment.

## 1. Introduction

A large amount of produced water is generated during oilfield development. Such produced water contains high concentrations of calcium, magnesium, and sulfate ions, which easily form scales in pipelines, leading to pipeline blockage, equipment corrosion, frequent workovers, and increased production costs [1,2,3]. Traditional water softening methods include chemical and physical processes. The most common chemical approaches are the “lime-soda process” and “caustic soda-sodium carbonate process”, which remove Ca^2+^ and Mg^2+^ by converting them into CaCO_3_ and Mg(OH)_2_ precipitates through dosing reagents such as lime (CaO) and soda ash (Na_2_CO_3_). These classic processes for oilfield wastewater softening can achieve 80–90% removal of Ca^2+^ and Mg^2+^, but suffer from drawbacks like high reagent costs, large sludge yields, and a high risk of secondary scaling [4,5,6,7,8]. In recent years, a green softening method using CO_2_ as a precipitation regulator has emerged, where CO_2_ dissolves in water to generate CO_3_^2−^, which reacts with Ca^2+^ to form CaCO_3_ precipitates, and pH regulation is further employed to optimize softening efficiency. In the treatment of coalbed methane produced water in Changqing Oilfield, a CO_2_ microbubble aeration process coupled with pH gradient control achieved a Ca^2+^ retention rate of 99.21% under conditions of raw water pH = 11.0, aeration rate = 200 mL/min, and adjusted pH = 12.8, with the effluent Ca^2+^ concentration meeting the reuse requirement for fracturing fluids [9,10,11]. However, this technology relies on the availability of a nearby CO_2_ source and requires massive alkali addition for pH adjustment, which significantly limits its widespread application.

Physical methods with lower environmental demands have attracted increasing attention. Mechanical vapor recompression (MVR) evaporation is a physical water treatment technology, but it is energy-intensive and prone to equipment scaling and corrosion. Ion exchange removes Ca^2+^ and Mg^2+^ via the selective adsorption of ion exchange resins, but its small treatment capacity cannot accommodate the large volume of discharged water from oilfields [12]. In contrast, nanofiltration (NF) membrane softening technology has been widely used in boiler water softening due to its excellent separation performance and chemical stability. Nevertheless, oilfield wastewater has a more complex composition than boiler water, making it more prone to scaling and fouling of NF membranes. To date, studies on the application of NF technology to treat oilfield wastewater remain limited [13,14].

In this study, a negatively charged NF membrane was prepared via interfacial polymerization. Its separation performance for Ca^2+^, Mg^2+^, and SO_4_^2−^ was systematically studied, and the membrane was applied to treat real produced water from the No. 1 Oil Production Plant of Xinjiang Oilfield. The separation mechanism and operating parameter effects were revealed, providing a new approach and technical support for low-cost and high-efficiency softening of high-salinity oilfield wastewater.

## 2. Materials and Methods

### 2.1. Materials and Instruments

The materials required for the experiment are shown in Table 1. The instruments used in the experiment included a GL2204C electronic analytical balance supplied by Beijing Zhongheng Rixin (Beijing, China), an SHA-CA water bath oscillator supplied by Beijing Yongmingguang (Beijing, China), an FL-43 diaphragm pump supplied by Fujian Baida (Xiamen, China), and an Orion Star^TM^ A221 water quality analyzer supplied by Thermo Fisher Scientific (Waltham, MA, USA).

### 2.2. Characterization

In order to better reflect the structural properties of the membranes used in the experiments, as well as to reflect the retention effect of each inorganic salt, we performed specific surface and porosity (BET), scanning electron microscopy (SEM), infrared spectroscopy (FTIR), and X-ray photoelectron spectroscopy (XPS) analyses on them. The relevant instruments used were the ASAP 2460 porosity analyzer from McMurray Tick Instruments, the Sigma 300 scanning electron microscope from ZEISS, Germany, the Thermo Scientific Nicolet iS20 infrared spectrometer from Thermo Electron, USA, and the Thermo Scientific K-Alpha X-ray photoelectron spectrometer. The concentrations of various ions were measured by ion chromatography. (Thermo Dionex ICS-6000, Waltham, MA, USA). The Zeta potential was measured using Malvern Zetasizer Ultra (Malvern Panalytical, Malvern, UK).

### 2.3. Experimental Setup

The synthesis process of the nanofiltration membrane is shown in Reference [15]:

(1) Fix the polysulfone support layer that has been rinsed with deionized water at the bottom of the plate frame (15 × 15 cm^2^), with the front side facing up, and use a rubber roller to remove the excess water droplets on the membrane surface.

(2) Pour 50 mL of a 1.5% aqueous solution of PIP-COOH onto the surface of the support layer, and set the contact time to 2 min to ensure that the base membrane is completely wetted by the aqueous solution of PIP-COOH. Then, quickly drain the aqueous phase solution and remove the remaining liquid.

(3) Add 30 mL of a 0.45% TMC/n-hexane solution to the polysulfone support layer coated with the PIP-COOH monomer, and let it react for 1 min to form an initial PA layer. Then, remove the excess TMC solution. Pour 30 mL of an isopropanol solution of polyethylenepolyamine with a mass fraction of 2% onto the membrane surface for amination. After keeping it for 1 min, remove the excess solution. Finally, carry out thermal curing in an oven at 65 °C for 5 min.

**Figure 1:**
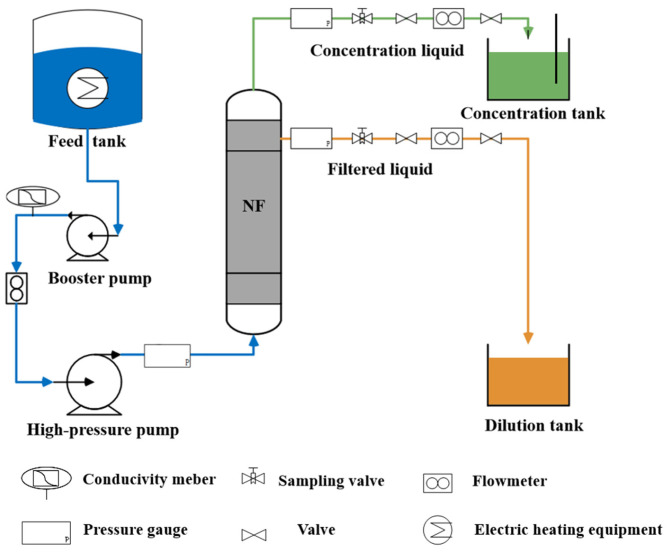
Nanofiltration experimental system.

The flow of this experimental setup is shown in Figure 1. After setting up the nanofiltration device, deionized water was first used to circulate in the nanofiltration system for 1–2 h, and compaction was carried out to ensure the stability of the membrane. The TDS of each solution was measured by a water quality analyzer, and the TDS of a single inorganic salt solution was used to reflect its inorganic salt content.

### 2.4. Experimental Parameters

Membrane flux is one of the key parameters used to characterize the membrane performance. The permeate flux of a nanofiltration membrane refers to the volume of filtrate that passes through a unit of membrane area in a unit of time, and its calculation formula is

(1) $$J=\frac{V}{S\times t}$$

where J is the permeate flux (L/m^2^∙h), t is the time of filtration (h), V is the volume of liquid permeated in time (L), and S is the unit membrane area (m^2^).

The retention rate of the nanofiltration membrane on solute can directly reflect the good or bad retention effect. Its calculation formula is

(2) $$R(\%)=(\frac{C_{f}-C_{p}}{C_{f}})\times 100$$

where R is the retention rate (%), C_f_ is the concentration of inorganic salts in the feed solution (mg/L), and C_p_ is the concentration of inorganic salts in the filtrate (mg/L).

Calcium Scaling Index (SI) and Scaling Aggressiveness Index (SAI) are widely used indicators to evaluate the calcium carbonate scaling tendency of water. The SI reflects the saturation state of calcium carbonate and predicts the likelihood of calcium deposition. A positive SI value indicates that the water is supersaturated with calcium carbonate and has a scaling risk; a negative SI value means the water is unsaturated and has no scaling tendency. The SAI further characterizes the comprehensive scaling and corrosive propensity of produced water. A higher positive SAI value represents a more severe scaling trend, while a lower or negative value indicates a weakened scaling risk. These indices are critical for assessing the reinjection suitability of oilfield produced water.

The molecular weight cut-off (MWCO) of the thin-film composite nanofiltration membrane was characterized using PEG solutions with a concentration of 1 g/L and molecular weights of 200, 400, 600, 800, 1000, and 2000 Da, respectively. The PEG molecular weight corresponding to a rejection of 90% was defined as the MWCO of the membrane. The detailed procedure is described in Reference [16].

### 2.5. Experimental Design

In the first experiment, the effect of different ion concentrations on the nanofiltration rejection performance was investigated. Altogether, 250, 750, 1250, 1750, and 2250 mg of solid CaCl_2_ were weighed and dissolved in 1 L of deionized water, respectively. After complete dissolution, CaCl_2_ aqueous solutions with concentrations of 250, 750, 1250, 1750, and 2250 mg/L were prepared. Na_2_SO_4_ and MgCl_2_ solutions were prepared using the same procedure. The solutions were passed through the nanofiltration membrane element at an inlet pressure of 0.4 MPa, an inlet temperature of 30 °C, and a filtration time of 2 min. The concentrations of Ca^2+^, SO_4_^2−^, and Mg^2+^ were measured respectively.

In the second experiment, the effect of operating pressure on the nanofiltration rejection performance of different inorganic salts was studied. The feed concentration was kept constant, and the operating pressure was adjusted by a control valve. CaCl_2_, Na_2_SO_4_, and MgCl_2_ solutions were filtered through the nanofiltration membrane element at inlet pressures of 0.30, 0.35, 0.40, 0.45, and 0.50 MPa for 2 min, respectively. The concentrations of Ca^2+^, SO_4_^2−^, and Mg^2+^ were determined respectively.

In the third experiment, produced water from the No. 1 Oil Production Plant of Xinjiang Oilfield was used as the treatment target. The filtration pressure was set at 0.4 MPa, and the changes in divalent ion concentrations and scaling tendency before and after treatment were investigated.

In the fourth experiment, tests were performed using produced water from the No. 1 Oil Production Plant at a fixed pressure of 0.4 MPa, with oil concentrations of 10 mg/L, 20 mg/L, 40 mg/L, 50 mg/L, and 100 mg/L, respectively.

The fifth experiment was conducted to evaluate the long-term stability. The ion retention rate and membrane flux were measured after 1, 10, 20, and 50 filtration cycles (20 min per cycle) during nanofiltration treatment of produced water, at 1000 mg/L CaCl_2_, 1000 mg/L MgCl_2_, and 500 mg/L Na_2_SO_4_ solutions, respectively.

## 3. Results and Discussion

### 3.1. Characterization Results

**Figure 2:**
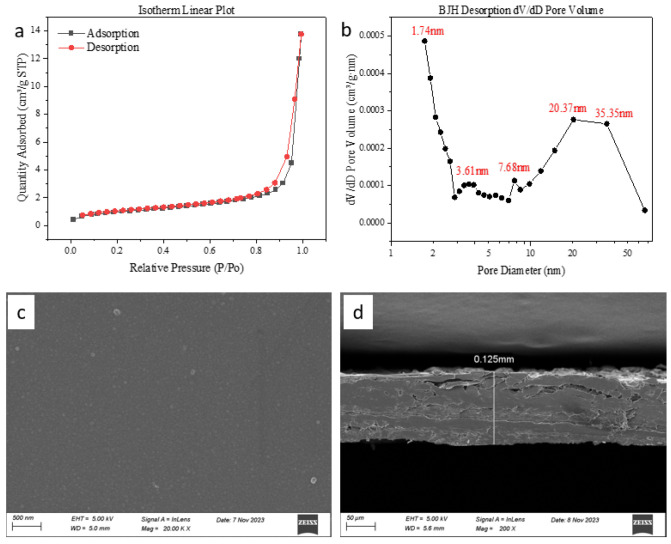
N_2_ adsorption–desorption isotherm of the membrane (**a**) and pore size distribution (**b**). SEM image of membrane surface (**c**). Cross section (**d**).

Figure 2a,b show the BET analysis curve of the pristine membrane, which indicates that the N_2_ adsorption–desorption isotherm is the type III isotherm. The isotherm is concave and has no inflection point. The amount of adsorbed gas rises with the increase in component partial pressure. The concave curve is caused by the interaction between adsorbate molecules being stronger than that between adsorbate and adsorbent, and the heat of adsorption in the first layer is smaller than the heat of liquefaction of adsorbate, so that adsorbate is more difficult to adsorb in the early stage of adsorption, and with the adsorption process, the adsorption appears to be a self-accelerating phenomenon, and the number of adsorbate layers is also not limited [17]. The BET surface area calculated from the N_2_ adsorption–desorption isotherm was 3.5835 m^2^/g. Scanning electron microscopy results are shown in Figure 2c,d; the original film sheet is characterized by a smooth, uniform and dense surface, uncontaminated, and the thickness of the film sheet is about 0.125 mm. The zeta potential of the membrane is −20.1 mV and the molecular weight cut-off (MWCO) of the nanofiltration membrane is approximately 400 Daltons.

**Figure 3:**
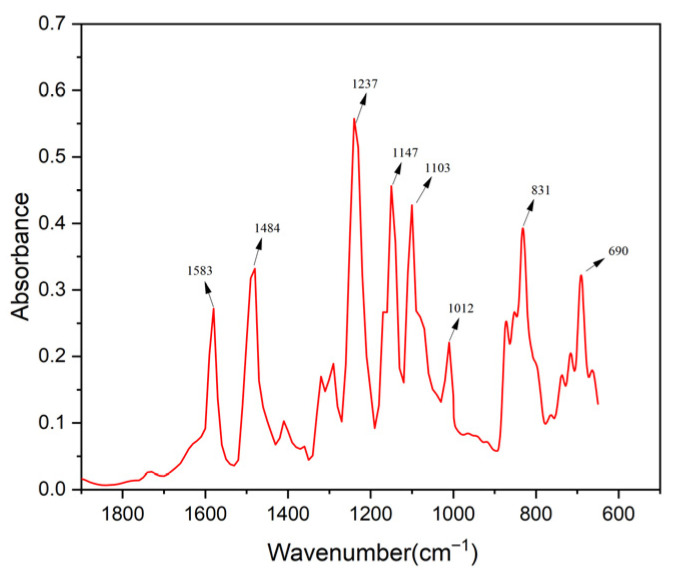
FTIR spectrum of the membrane.

Figure 3 shows the Fourier-transform infrared spectroscopy (FTIR) of the sample. The peaks at 690 cm^−1^ and 831 cm^−1^ may correspond to the C-H bending vibration of the aromatic ring; the peaks at 1012 cm^−1^ and 1130 cm^−1^ may correspond to the acyl (C=O) stretching vibration of polyamide; the peak at 1237 cm^−1^ may correspond to the N-H bending vibration of amide; and the peak at 1484 cm^−1^ may correspond to the C-N stretching vibration of aromatic amine. The peak at 1583 cm^−1^ may correspond to the C=C stretching vibration of aromatic rings [18].

**Figure 4:**
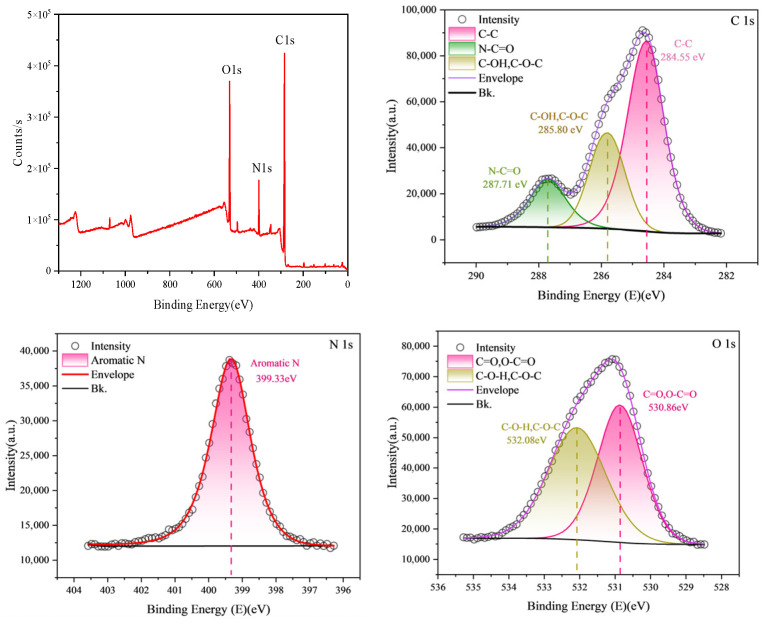
The full spectrum of XPS and the fine spectrum of C1s, N1s and O1s.

X-ray photoelectron spectroscopy (XPS) results (Figure 4) indicate that the membrane surface mainly consists of C, O, and N with contents of 68.21%, 19.49%, and 8.95%, respectively. C 1s spectra can be deconvoluted into N–C=O (287.71 eV), C–OH/C–O–C (285.8 eV), and C–C (284.55 eV), and O 1s into C–OH/C–O–C (532.08 eV) and C=O/O–C=O (530.86 eV); N 1s is dominated by aromatic nitrogen (399.33 eV), consistent with FTIR results [19].

### 3.2. Performance Tests

**Figure 5:**
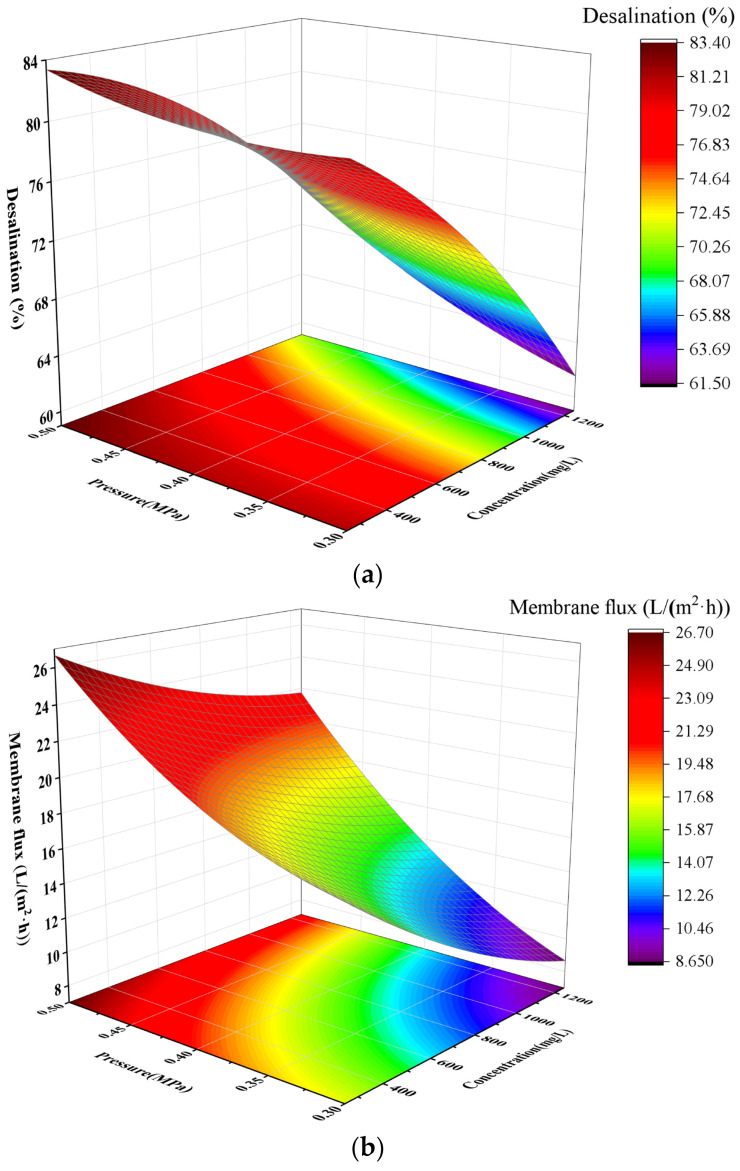
The effects of calcium ion concentration and pressure on desalting rate (**a**) and membrane flux (**b**).

Figure 5a shows the calcium removal efficiency of the nanofiltration membrane for calcium chloride at different concentrations and pressures, and Figure 5b presents the flux of the nanofiltration membrane under the same conditions. It can be observed that at a constant concentration of 250 mg/L, the calcium removal efficiency increased slightly with the rise in pressure, accompanied by a significant increase in membrane flux. With the increase of calcium chloride concentration from 250 mg/L to 750 mg/L, the calcium removal efficiency decreased from 82% to 72%, and the flux also dropped from 26 L/m^2^·h to 23 L/m^2^·h. When the concentration further increased to 1250 mg/L, the calcium removal efficiency declined to 62% and the flux decreased slightly from 23 L/m^2^·h to 22 L/m^2^·h. Nevertheless, at this concentration, the calcium removal efficiency still rose slightly and the flux increased significantly with the elevation of pressure. Overall, the increase in operating pressure exerts a remarkable effect on improving both the desalination efficiency and membrane flux. The underlying mechanisms are as follows:

(1) With the increase in pressure, the water flux increases substantially due to the small molecular volume of water molecules [20,21]. In contrast, the permeation rate of calcium ions rises at a much lower rate owing to the electrostatic repulsion and sieving hindrance of the membrane. A large number of water molecules pass through the membrane pores rapidly, which dilutes the salt ion concentration on the membrane surface, alleviates the concentration polarization phenomenon, and reduces the mass transfer of salt ions to the permeate side, driven by the concentration difference, thus enhancing the desalination efficiency.

(2) There exists a concentration difference-driven reverse diffusion of salt ions across the membrane [22]. When the pressure increases, the salt concentration on the permeate side is rapidly diluted, while the salt concentration on the membrane surface shows a negligible increase because water molecules carry away the surface salt ions quickly. This ultimately narrows the salt concentration difference between the two sides of the membrane, leading to a significant reduction in the driving force for the reverse diffusion of salt ions. As a result, the salt permeation rate decreases and the desalination efficiency is improved.

(3) The electric double layer on the surface of the nanofiltration membrane occupies part of the effective space of membrane pores [23]. An increase in pressure causes slight compressive deformation of the porous structure of the membrane, which reduces the effective pore size slightly and simultaneously increases the relative density of fixed charges on the membrane surface. This change brings about two effects. (a) Enhanced sieving effect: The reduced effective pore size results in a more significant physical retention of calcium ions, as shown in Table 2. (b) Enhanced Donnan repulsion: The higher surface charge density generates a stronger electrostatic repulsion, which exerts a greater hindering effect on salt ions with the same charge. Even for low-valence salt ions (e.g., Na^+^, Cl^−^), it becomes more difficult for them to cross the electric double layer and enter the membrane pores [24].

**Figure 6:**
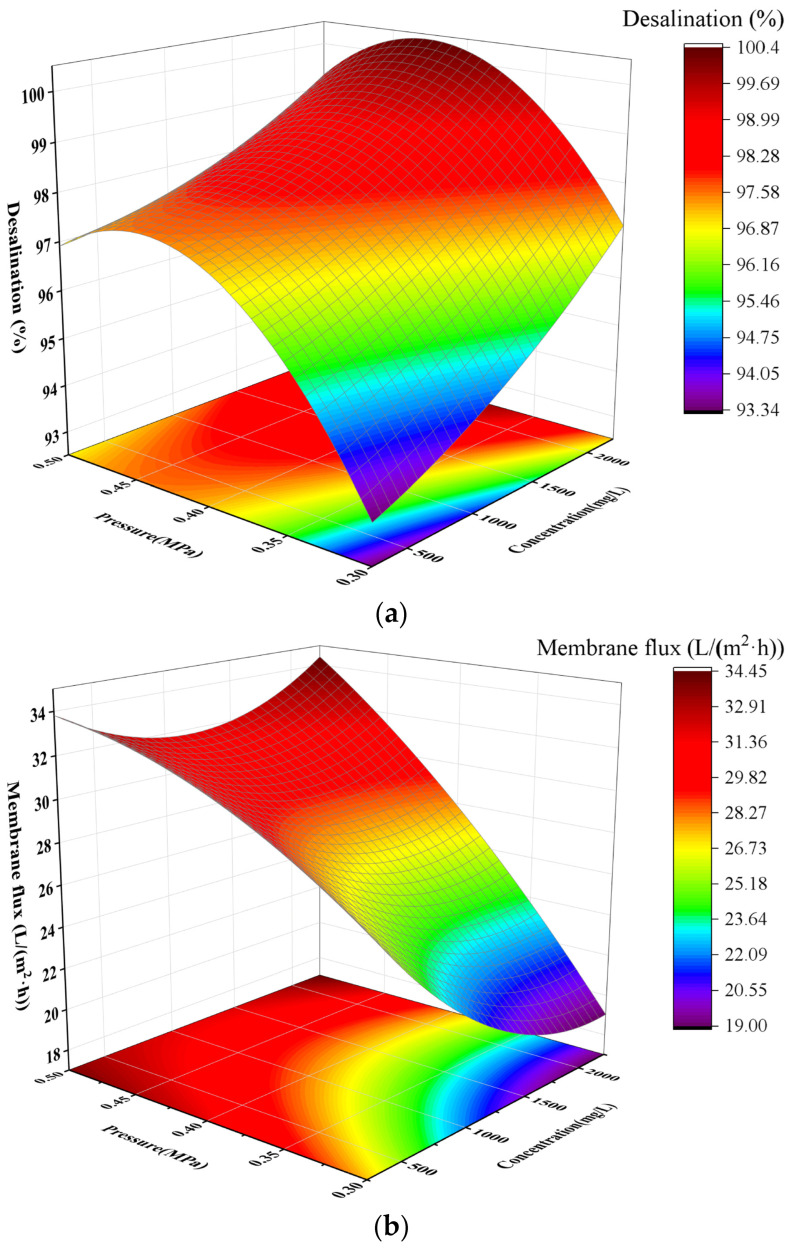
The effects of sulfate ion concentration and pressure on removal efficiency (**a**) and membrane flux (**b**).

**Figure 7:**
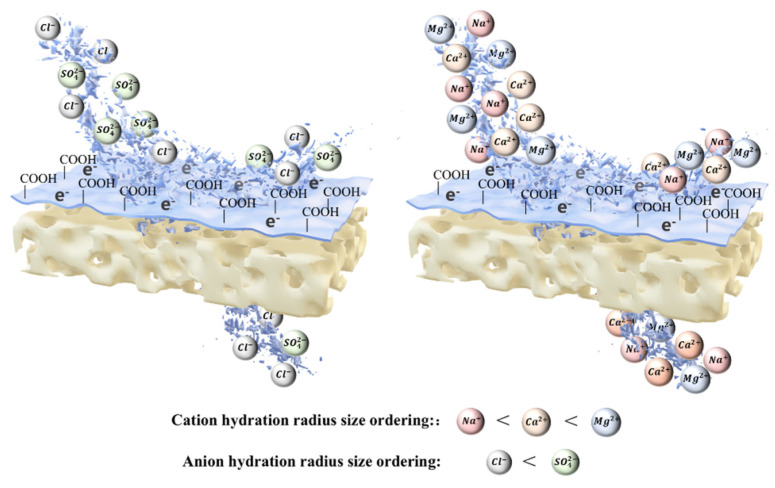
Schematic diagram of different cations and ions trapped by polyamide membrane.

Figure 6a presents the sulfate removal efficiency of the nanofiltration membrane for sodium sulfate under different concentrations and pressures, and Figure 6b shows the membrane flux under the same conditions. It can be observed that at a constant concentration of 250 mg/L, the sulfate removal efficiency reached 94% initially and increased slightly with the rise in pressure, with a simultaneous significant increase in membrane flux. As the sodium sulfate concentration increased from 250 mg/L to 1250 mg/L, the sulfate removal efficiency rose from 96% to 99%, while the flux decreased from 34 L/m^2^·h to 31 L/m^2^·h. When the concentration further increased to 2250 mg/L, the sulfate removal efficiency remained stable at 99%, and the flux recovered to 34 L/m^2^·h. Overall, the nanofiltration membrane exhibited a much higher removal efficiency for sulfate ions than for calcium ions. This is because the charging properties of nanofiltration membrane materials have different selective separation performances for different salt systems, in addition to the size of the ion hydration radius and diffusion coefficient also determining the retention performance of nanofiltration membranes [25], as shown in Figure 7. Due to the polyamide membrane surface with a large number of carboxyl groups (-COOH) being negatively charged, the anion rejection effect is large, so it makes it easier for the nanofiltration membrane to retain the anion than the cation [26], and with SO_4_^2−^ having more charges than Cl^−^, the ionic radius is larger than Cl^−^, so nanofiltration on the Na_2_SO_4_ retention effect is better than CaCl_2_.

**Figure 8:**
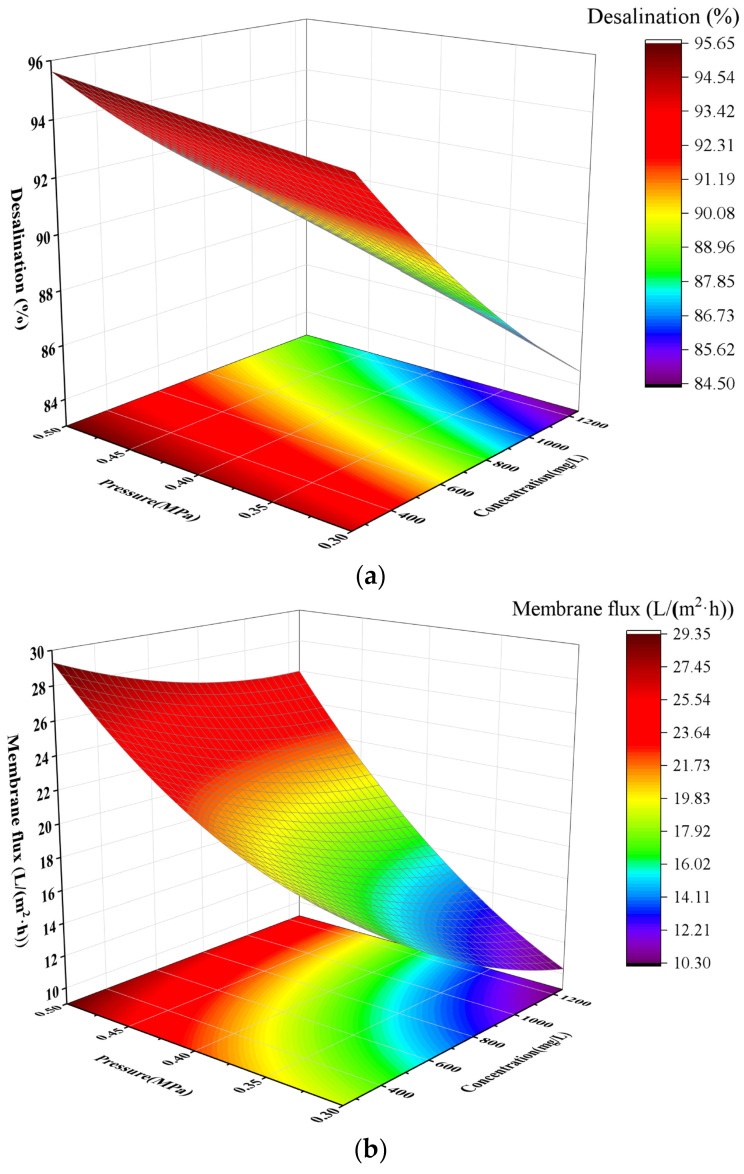
The effects of magnesium ion concentration and pressure on desalting rate (**a**) and membrane flux (**b**).

Figure 8a shows the magnesium removal efficiency of the nanofiltration membrane for magnesium chloride under different concentrations and pressures, and Figure 8b presents the membrane flux under the same conditions. It can be observed that at a constant concentration of 250 mg/L, the magnesium removal efficiency reached 94% initially and increased slightly with the rise in pressure, accompanied by a significant increase in membrane flux. As the magnesium chloride concentration increased from 250 mg/L to 750 mg/L, the magnesium removal efficiency decreased from 94% to 87%, and the flux dropped from 18 cm^3^/min to 14 cm^3^/min. When the concentration further increased to 1250 mg/L, the magnesium removal efficiency declined to 85%, with the flux remaining at 14 cm^3^/min. Overall, the nanofiltration membrane exhibited a higher removal efficiency for magnesium ions than for calcium ions. The underlying mechanism is as follows: The bare ionic radius of magnesium ions is 0.072 nm, which is smaller than that of calcium ions (0.100 nm). However, magnesium ions have a much higher charge density than Ca^2+^, leading to a stronger ability to polarize and adsorb water molecules. Their hydrated particle size reaches 4.3 nm, which far exceeds the upper limit of the nanofiltration membrane pore size. In contrast, the hydrated particle size of calcium ions is approximately 2.6 nm, close to the upper limit of the membrane pore size, allowing partial passage through the membrane pores with smaller sizes. This constitutes the key reason for the higher removal efficiency of magnesium ions [27,28,29,30].

**Figure 9:**
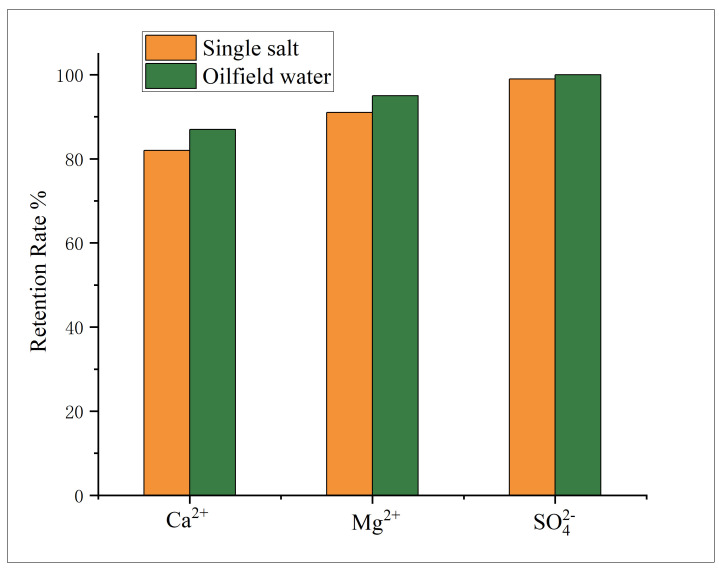
Effect of different inorganic salt operating pressures on membrane flux.

The water quality of produced water from the No. 1 Oil Production Plant of Xinjiang Oilfield is shown in Table 3. It can be seen that the concentrations of Ca^2+^ and Mg^2+^ are as high as 1000 mg/L, and the SO_4_^2−^ concentration reaches 420 mg/L. The SI and SAI values are up to 4.4 and 3.2, respectively, indicating an extremely severe scaling tendency. Figure 9 presents the retention rate of divalent ions after treatment by the nanofiltration membrane at 0.4 MPa. It is clear that the removal rates of Ca^2+^, Mg^2+^, and SO_4_^2−^ are 91%, 95%, and 100%, respectively, which are significantly improved compared with those in single-salt solutions. The effluent scaling index (SI) is −0.6 and the scaling inhibition index (SAI) is 8.5, indicating complete elimination of the scaling tendency. This is mainly attributed to the enhanced complexation among various ions in real produced water, which substantially increases the effective hydration radius and thus leads to higher rejection than in single-salt systems [31,32,33].

**Figure 10:**
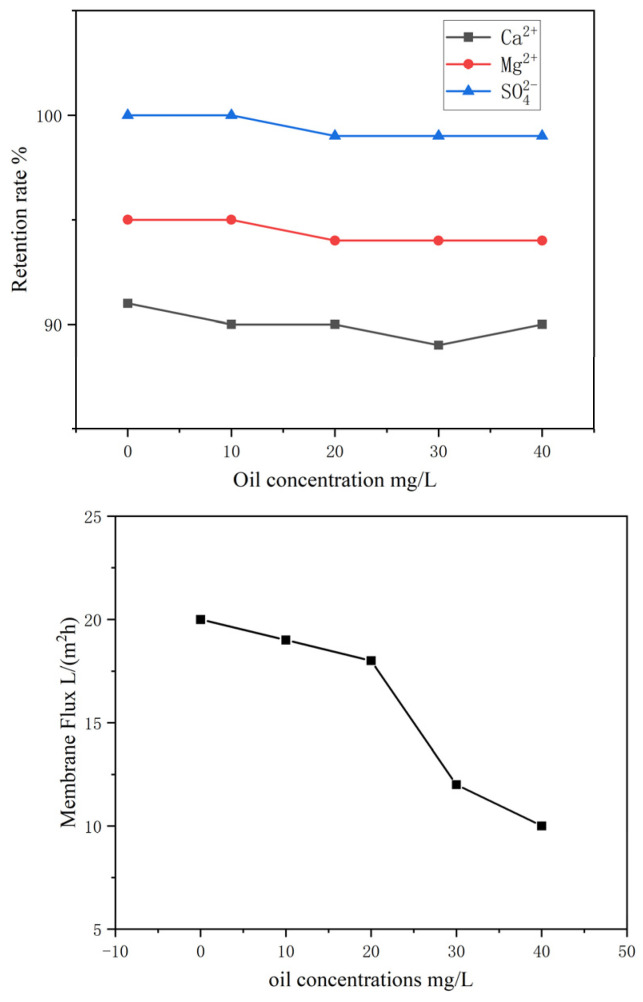
Effect of oil concentration on retention rate and membrane flux.

Figure 10 shows the ion retention rate and membrane flux of crude oil produced water at different oil concentrations. It can be seen that with the increase in oil content, the ion retention rate remains basically unchanged, while the membrane flux decreases. When the oil concentration exceeds 30 mg/L, the membrane flux drops sharply. This is mainly because a large number of large-sized petroleum emulsions are formed above 30 mg/L, which block the membrane pores and lead to a significant decline in flux.

### 3.3. Scanning Electron Microscopy Analysis of Post-Filtration Membranes

**Figure 11:**
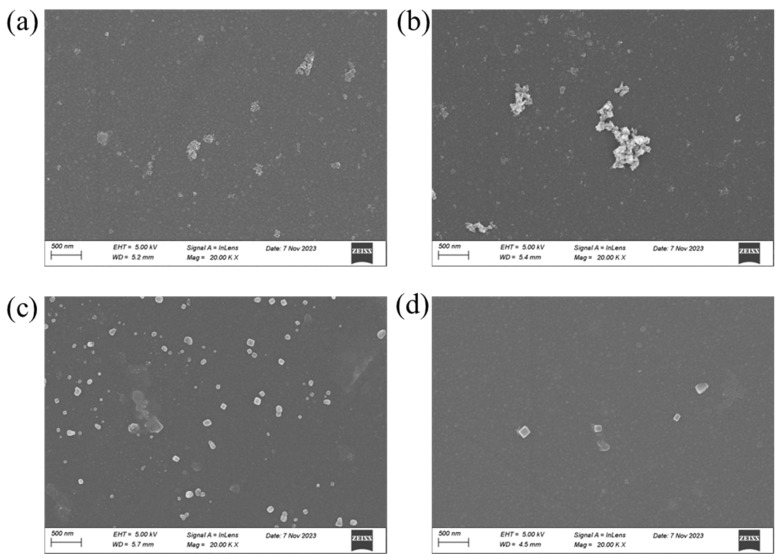
(**a**–**d**) SEM images of the membrane surface after product water, pure Na_2_SO_4_, CaCl_2_ and MgCl_2_ were trapped by the nanofiltration membrane.

Figure 11 displays the SEM images of the nanofiltration membrane after treatment of oilfield produced water, pure Na_2_SO_4_, pure CaCl_2_, and pure MgCl_2_ solutions, respectively. Clearly visible crystal precipitates are observed on the membrane surface. Specifically, the membrane used for treating oilfield produced water exhibits crystal grains of 200–300 nm with a small number of clusters. The membrane exposed to sodium sulfate shows the largest aggregates, reaching up to 2 μm. The membrane treated with calcium chloride presents abundant crystals ranging from 100 to 200 nm. In comparison, the membrane processed with magnesium chloride displays much finer crystallites of approximately 100 nm. The main reason lies in the different crystalline morphologies: CaCl_2_ crystals mainly belong to the cubic system, characterized by distinct edges, compact structure, and relatively large size, which readily form a continuous and dense fouling layer. By contrast, MgCl_2_ crystals are mostly hexagonal or monoclinic, featuring slender, well-dispersed structures with loose distribution [34].

**Figure 12:**
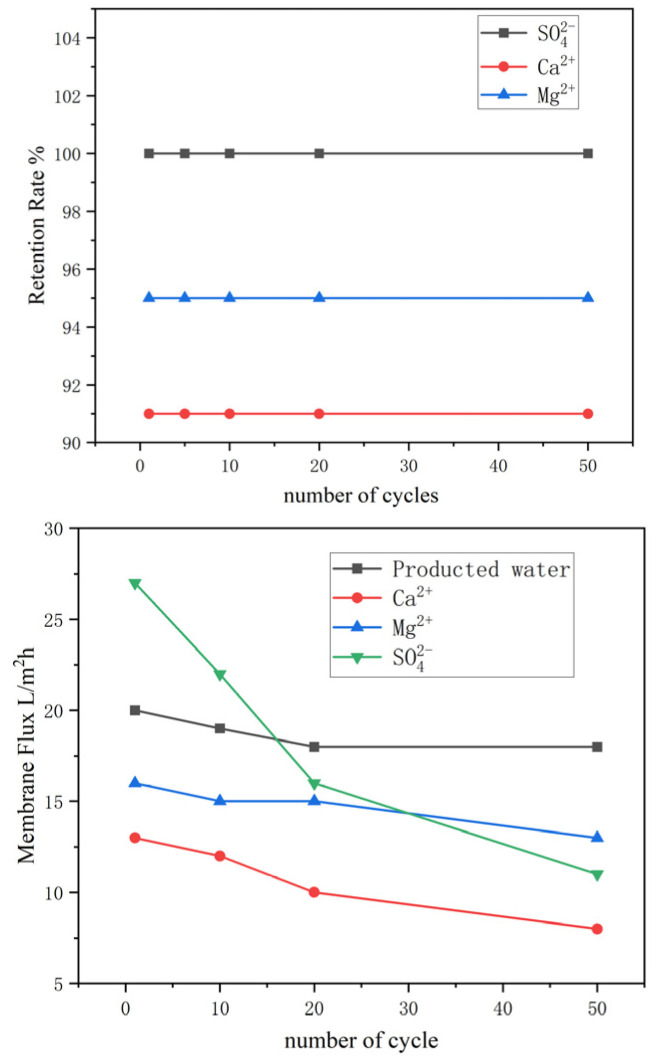
Retention rate and membrane flux of oilfield produced water, pure CaCl_2_, MgCl_2_, and Na_2_SO_4_ after 1, 10, 20, and 50 filtration cycles.

Figure 12 shows the ion retention rate and membrane flux of oilfield produced water, pure CaCl_2_, MgCl_2_, and Na_2_SO_4_ solutions after 1, 10, 20, and 50 filtration cycles. It can be seen that the ion removal efficiency remains almost unchanged, while the membrane flux gradually decreases with an increasing number of cycles. Among them, the flux decline is the fastest for sulfate ions, and the slowest for Mg^2+^ ions. This is mainly because the crystals formed by Mg^2+^ precipitation are the smallest and least abundant, thus causing the least damage to the membrane.

## 4. Conclusions

In this paper, a polyamide nanofiltration membrane was synthesized by the interfacial polymerization technique and its surface was aminated. The results show that the surface modification method significantly enhances the positive charge of the membrane and improves water permeation. The effect of treating a single inorganic salt with this membrane was studied. The results indicate that the membrane achieves a maximum rejection rate of 99% for the divalent anion sulfate ions (SO_4_^2−^), with maximum rejection rates of 81% and 94% for calcium ions (Ca^2+^) and magnesium ions (Mg^2+^), respectively. With the increase in the inorganic salt concentration, the Donnan effect of the membrane weakens, while the shielding effect strengthens; the membrane flux and the removal efficiency of target ions decrease accordingly. When this membrane was applied to the treatment of complex salt wastewater from No. 1 Oil Production Plant of Xinjiang Oilfield, the results showed that the removal efficiency of sulfate ions reached 100%, and those of calcium and magnesium ions were 91% and 95%, respectively. After treatment, the scaling indices SI and SAI of the water quality were −0.6 and 8.5, respectively, and the scaling tendency was completely eliminated. This process study lays a foundation for the application of nanofiltration technology in the field of oilfield wastewater treatment.

## Figures and Tables

**Table 1:** Materials.

Materials	Purity	Manufacturer
PIP-COOH	99%	Aladdin Reagent (Shanghai, China)
TMC	99%	Aladdin Reagent
Polyethylene Polyamine	99%	Aladdin Reagent
NaCl	99%	Sinopharm Chemical Reagent Co., Ltd. (Beijing, China)
Na_2_SO_4_	99%	Sinopharm Chemical Reagent Co., Ltd.
CaCl_2_	99%	Sinopharm Chemical Reagent Co., Ltd.
MgCl_2_	99%	Sinopharm Chemical Reagent Co., Ltd.
Hexylhydride	99%	J&K Scientific Reagent (Beijing, China)
Isopropyl alcohol	99%	J&K Scientific Reagent
Oilfield wastewater		Xinjiang Oilfield (Xinjiang, China)
PEG	99%	Sinopharm Chemical Reagent Co., Ltd.

**Table 2:** Hydrated ionic radius and ion diffusion coefficient [24].

Ionic	Hydrated Ionic Radius/nm	Diffusion Coefficient/(10^−9^ m^2^·s^−1^)
Na^+^	0.358	1.33
Ca^2+^	0.413	0.92
Mg^2+^	0.428	0.72
Cl^−^	0.332	2.03
SO_4_^2−^	0.397	1.06

**Table 3:** Ion distribution in the water of the First Oil Production Plant and the scaling indexes SI and SAI.

Ca^2+^ mg/L	Mg^2+^ mg/L	HCO_3_^−^ mg/L	SO_4_^2+^ mg/L	Cl^−^ mg/L	Na^+^ mg/L	SI	SAI
1000	1000	420	490	6200	3100	4.4	3.2

## Data Availability

The original contributions presented in this study are included in the article. Further inquiries can be directed to the corresponding author.
